# Porous Materials Based on Poly(methylvinylsiloxane) Cross-Linked with 1,3,5,7-Tetramethylcyclotetrasiloxane in High Internal Phase Emulsion as Precursors to Si-C-O and Si-C-O/Pd Ceramics

**DOI:** 10.3390/ma14195661

**Published:** 2021-09-29

**Authors:** Jan Mrówka, Janusz Partyka, Magdalena Hasik

**Affiliations:** 1 Department of Silicate Chemistry and Macromolecular Compounds, Faculty of Materials Science and Ceramics, AGH University of Science and Technology, al. Mickiewicza 30, 30-059 Kraków, Poland; mhasik@agh.edu.pl; 2 Department of Ceramics and Refractory Materials, Faculty of Materials Science and Ceramics, AGH University of Science and Technology, al. Mickiewicza 30, 30-059 Kraków, Poland; partyka@agh.edu.pl

**Keywords:** polysiloxanes, polymer-derived ceramics, silicon oxycarbide, polyHIPE

## Abstract

Polysiloxane networks were prepared by hydrosilylation of poly(methylvinylsiloxane) (V_3_ polymer) with 1,3,5,7-tetramethylcyclotetrasiloxane (D_4_^H^) at various Si-Vinyl: Si-H groups molar ratios in water-in-oil high internal phase emulsion (HIPE). Curing the emulsions followed by removal of water led to foamed cross-linked polysiloxane systems differing in the cross-linking degrees, as well as residual Si-H and Si-Vinyl group concentrations. Treatment of thus obtained materials in Pd(OAc)_2_ solution in tetrahydrofuran resulted in the formation of porous palladium/polymer nanocomposites with different Pd contents (1.09–1.70 wt %). Conducted investigations showed that pyrolysis of the studied materials at 1000 °C in argon atmosphere leads to porous Si-C-O and Si-C-O/Pd ceramics containing amorphous carbon and graphitic phases. Thermogravimetric (TG) analysis of the starting cross-linked polymer materials and those containing Pd nanoparticles revealed that the presence of palladium deteriorates thermal stability and decreases ceramic yields of preceramic networks. The extent of this effect depends on polymer cross-linking density in the system.

## 1. Introduction

Polysiloxanes, which are represented by the general formula of (RR’SiO)_n_, are a class of hybrid organic/inorganic polymers. Their main chain consists of repeating Si-O bonds, which are longer and more flexible than the C-C ones forming the backbones of common organic polymers. This structural difference results in many interesting properties of polysiloxane materials, such as high gas permeability, low melting point, and high thermal stability [1].

Pyrolysis of cross-linked polysiloxanes in an inert atmosphere leads to various ceramics, such as amorphous Si-C-O materials [2,3] or crystalline SiC (after etching of the pyrolyzed materials with HF) [4], making polysiloxanes a class of valuable preceramic polymers. Amorphous Si-C-O, also called silicon oxycarbide materials, consist of SiO_x_C_4−x_ units, with ‘x’ ranging from 0 to 4 [3]. They have been an area of interest for many years due to their many outstanding properties as compared to pure silica glass, such as improved thermal and chemical stability, good mechanical properties, and high creep resistance to name just a few. It has been long established that these enhanced properties are due to carbon substitution for oxygen atoms in SiO_4_ tetrahedra of silica [5,6].

Si-C-O materials cannot be prepared by classic melting of raw materials—i.e., silicon dioxide and carbon—because of many side reactions that take place at high temperatures. Preceramic polymers allow to obtain silicon oxycarbide glasses at relatively low temperatures (1000 °C) which prevents them from thermal degradation [7]. The main parameter that influences ceramic yield of the polysiloxane systems is their cross-linking degree. Heating of the linear, uncross-linked poly(dimethylsiloxane) (PDMS)—which is the most commonly applied polysiloxane—in an inert gas leads to no residue, because of its decomposition to volatile cyclic oligomers [8]. Presence of oxygen in the furnace’s atmosphere causes PDMS to cross-link which impedes formation of cyclosiloxanes and the material decomposes to solid SiO_2_ and gaseous products: CO_2_, H_2_O [8].

Several methods, including condensation chemistry [9], use of organoperoxides [10,11], and hydrosilylation [4,10,12] can be applied to efficiently cure polysiloxanes. Particularly interesting preceramic polysiloxane-based systems are those obtained by cross-linking of linear polymers with cyclic compounds. Existence of cyclic moieties in preceramic polymer structures, due to their high thermal stability, is known to ensure high yield of ceramics [12,13]. Moreover, the use of multifunctional cyclic cross-linking agents leads to relatively high cross-linking degrees of the polymer with preservation of significant amounts of functional groups of the cross-linker in the network. These groups can be used as reactive sites in the processes performed after polymer cross-linking to modify the preceramic material which, upon pyrolysis, yields ceramics of the desired composition and/or properties. For example, in our previous work it was shown that Si-H groups remaining in poly(vinylsiloxane) reduce Pd^2+^ and Pt^4+^ ions to their metallic forms [14,15]. Pyrolysis of these materials results in Si-C-O ceramics containing Pd or Pt particles. It was also found that the presence of Pd or Pt in the preceramic systems influences the mechanism of their thermal degradation and increases ceramic yields [14,15]. Catalytic conversion of propan-2-ol conducted in the presence of both, preceramic poly(vinylsiloxanes) and Si-C-O materials with introduced Pt particles was studied [15]. It should be noted that the described studies concerned preceramic networks of non-porous microstructure. However, in some (e.g., catalytic) applications, porosity of the materials could be advantageous. When both presence of metallic particles and high thermal stability are required, the use of porous Si-C-O ceramics–metal systems should be preferred.

Although there are many studies devoted to polymer-derived silicon oxycarbide materials, only few of them address macrocellular Si-C-O monoliths. Several methods were used to produce macrocellular Si-C-O monolithic ceramics showing various types of porosity—e.g., preceramic polysiloxane material was foamed, and mesoporous organosilica [16] or poly(methyl methacrylate) microbeads [17] were used to introduce porosity. There are also some recently published papers describing the pyrolysis of poly(methylhydrosiloxane) cross-linked by hydrosilylation, in which cellular morphology is obtained by addition of hydrotalcite during cross-linking [18] or H_2_ evolution and PDMS sacrificial loss [19].

In this work, we obtained macrocellular Si-C-O and Si-C-O/Pd materials using a new approach. First, polysiloxane networks (polysiloxane-based polyHIPEs) were prepared by hydrosilylation of poly(methylvinylsiloxane) (V_3_ polymer) with a cyclic hydrosiloxane, namely 1,3,5,7-tetramethylcyclotetrasiloxane (D_4_^H^) in high internal phase emulsion (HIPE), and to these materials palladium was incorporated. Both, Pd-free and Pd-containing materials were then pyrolyzed at 1000 °C in Ar atmosphere. It was found that the microstructure of these preceramics is preserved. The decomposition processes that take place during pyrolysis of the initial and Pd-containing networks were investigated by thermogravimetry (TG) to determine the influence of palladium presence on thermal stability and ceramic yield of the materials.

The work continues our previous studies in which V_3_ polymer-based networks obtained in HIPE with introduced Pd particles were used as catalysts of phenylacetylene hydrogenation [20]. The present investigations were primarily aimed at characterization of the pyrolyzed materials, as well as at characterization of thermal properties of the initial and Pd-containing systems, which are important for their pyrolysis.

The Pd-containing porous Si-C-O materials that are described in this paper can find applications—e.g., in the field of catalysis. The use of porous Si-C-O systems, containing Ni, Co, or Pt as catalysts was studied in the processes of CO_2_ methanation [21,22,23,24]. However, those papers describe materials in which porosity was introduced using other methods—i.e., either by sacrificial loss of additives in the system [21], direct foaming [23] or freeze casting [22]. According to our knowledge, there are no reports on using preceramic polyHIPEs to obtain porous Si-C-O ceramics or Si-C-O-Pd composite materials. This is, so far, the first report in the literature on using preceramic polyHIPEs to obtain macrocellular Si-C-O ceramics.

As it was reported in our previous work [20], most of polyHIPEs studied so far are based on organic polymers. They are the subject of many excellent reviews [25,26,27,28]. Various monomers and polymerization methods were used to form polyHIPEs. Only a few reports describe polyHIPEs prepared from polysiloxanes [20,29]. Polysiloxane-based polyHIPEs are of great interest, since as shown in this work they can be used as precursors to macroporous Si-C-O materials and Si-C-O/metal nanocomposites.

## 2. Materials and Methods

### 2.1. Starting Materials

V_3_ polymer was synthesized by anionic ring-opening polymerization of 1,3,5-trimethyl-1,3,5-trivinylcyclotrisiloxane (ABCR, Karlsruhe, Germany), according to the previously described procedure [14]. The average molecular weight (M_n_) and polydispersity index (M_w_/M_n_) of the obtained polymer were determined by gel permeation chromatography (GPC) (Malvern Panalytical-Viscotek, Houston, TX, USA) in dichloromethane using polystyrene standards, and were equal to 6800 g/mol and 1.2, respectively, while M_n_ obtained from ^1^H NMR spectra (Bruker Corp., Rheinstetten, Germany) was 6250 g/mol. Other chemicals used in the experiments were commercial products and are given, together with their purification methods, in [20].

### 2.2. Preparation of Polysiloxane, Polysiloxane-Pd Networks, and the Pyrolyzed Materials

V_3_ polymer/D_4_^H^ porous, monolithic materials (polyHIPEs) were obtained by cross-linking of V_3_ polymer with D_4_^H^ in water-in-oil (w/o) HIPE conditions in the way described in [20]. The continuous phase of HIPE contained the polymer, the cross-linking agent (D_4_^H^), the surfactant (DBE-224), additional porogen (chlorobenzene), and Karstedt hydrosilylation catalyst, whereas the internal phase was the 0.02M NaCl aqueous solution. The amounts of D_4_^H^ used were such that molar ratios of Si-Vinyl of the polymer to Si-H groups of D_4_^H^ in the experiments were equal to 2.25:1, 1.5:1, 1:1, and 1:1.5 for the samples denoted as 2.25V3_1Si-H, 1.5V3_1Si-H, 1V3_1Si-H, and 1V3_1.5Si-H, respectively. Detailed amounts of HIPE components used in the preparation of the studied polyHIPEs are given in Table 1.

Palladium was introduced into the prepared porous materials, after crushing, by their treatment with the 4.7 × 10^−3^ M Pd(OAc)_2_ solution in tetrahydrofuran following the procedure presented in our previous studies [20]. The amount of the solution used was such that—if all metal present in the solution was introduced into the polymer matrix—Pd would constitute 2.0 wt % of the material—i.e., 4.08 cm^3^ of the solution per 1 g of the polyHIPE material were applied. The materials treated in this way will be subsequently referred to as 2.25V3_1Si-H_Pd, 1.5V3_1Si-H_Pd, 1V3_1Si-H_Pd, and 1V3_1.5Si-H_Pd.

Pyrolysis of the samples was conducted in a quartz tube furnace in Ar atmosphere. Typically, 500 mg of the sample were placed in a graphite crucible and held at 1000 °C for 6 h. The heating rate was 5 °C/min. To denote the pyrolyzed materials, subscript ‘pyrolyzed’ will be added to the symbol of the sample subjected to pyrolysis. For example, 2.25V3_1Si-H_pyrolyzed_ and 2.25V3_1Si-H_Pd_pyrolyzed_ will mean the pyrolyzed 2.25V3_1Si-H and 2.25V3_1Si-H_Pd materials, respectively.

### 2.3. Characterization Methods

Raman spectra of the initial samples were recorded on a WITec alpha300M+ (WITec Wissenschaftliche Instrumente und Technologie GmbH, Ulm, Germany), using 488 nm laser and 1800 diffraction grating. The spectra of Pd-containing and pyrolyzed samples were measured on LabRAM HR (Horiba Scientific, Kyoto, Japan) microscope using a 532 nm laser for excitation and 1800 diffraction grating. The use of two spectrometers was necessary as the fluorescence of the samples made it impossible to perform the measurements with one laser excitation line. The mean carbon domain size was calculated from the general equation (Equation (1)) connecting crystallite size (L_a_) with integrated intensities of the D and G bands (I_D_/I_G_) in the Raman spectra and the excitation wavelength [30]

(1) $$La=2.4•10^{-10}•\lambda^{4}•{\left(\frac{I_{D}}{I_{G}}\right)}^{-1}\;$$

where *La* (nm) is a crystallite size, *λ* (nm) is a laser line wavelength and *I_D_* and *I_G_* stands for the integral intensity of D and G band in the Raman spectra, respectively.

FT-IR spectra were collected using attenuated total reflectance (ATR) method in the range of 550–4000 cm^−1^ with BIO-RAD FTS6000 (Bio-Rad, Hercules, CA, USA) spectrometer using a ZnSe crystal and 45° incident beam angle. Resolution of the measurements was equal to 4 cm^−1^.

X-ray diffractometry (XRD) data were collected by PANalytical Empyrean powder diffractometer (Malvern Panalytical, Almelo, The Netherlands) using Kα radiation from Cu anode. The transmission mode configuration with rotating sample was used. The primary beam setup consisted of focusing mirror and 1/32° molybdenum divergence slit. All measurements were carried out at room temperature and under ambient pressure. Mean crystallite sizes of Pd were calculated using Philips X’Pert High Score Plus software (version 3.0d (3.0.4), Malvern Panalytical, Almelo, The Netherlands). The Pd(111) reflex was fitted using Pseudo Voigt function and its full width at half maximum (FWHM) was used to quantify the mean crystallite size with Scherrer equation (Equation (2)).

(2) $$D=\frac{K•\lambda }{\beta •\cos\theta }\;$$

where;

*D*—mean crystallite size (nm)
K—Scherrer constant; in the performed calculations K = 1was applied
*λ*—wavelength of the X ray radiation, λ = 0.15406 nm for CuKα lamp
β—full width at half maximum (FWHM) of the Pd(100) peak (deg)
*θ*—Bragg diffraction angle (deg)

Thermogravimetric (TG) studies were conducted using a NETZSCH STA 449F3 apparatus (Netzsch GmbH, Selb, Germany) for up to 1000 °C with heating rate of 5 °C/min under Ar flow. Typically, 14 mg of the sample were used during thermal analysis.

Scanning Electron Microscope (SEM) observations of the samples without Pd were made on a Phenom XL scanning electron microscope (Thermo Fisher Scientific, Waltham, MA, USA) equipped with Energy Dispersive X-ray Spectroscopy (EDS) analyzer after being sputtered with 40 nm Au coat. EDS quantification was performed during measurements conducted in high vacuum using the standardless method. Raw data were corrected to account for absorption and fluorescence of X-rays in the computer program provided by the instrument producer. The default method, optimal for analysis of the samples especially in the low energy range, was applied.

SEM studies of the Pd-containing materials were performed using an ultra-high resolution scanning electron microscope (NOVA NANO SEM 200, FEI EUROPE COMPANY, Hillsboro, OR, USA) equipped with Schottky emitter (FEG) in the Back-Scattered Electron detection (BSED) mode. Prior to the examinations, the samples were sputtered with carbon for better conductivity.

## 3. Results and Discussion

### 3.1. V_3_ Polymer-Based PolyHIPEs before and after Pyrolysis

**Scheme 1:**
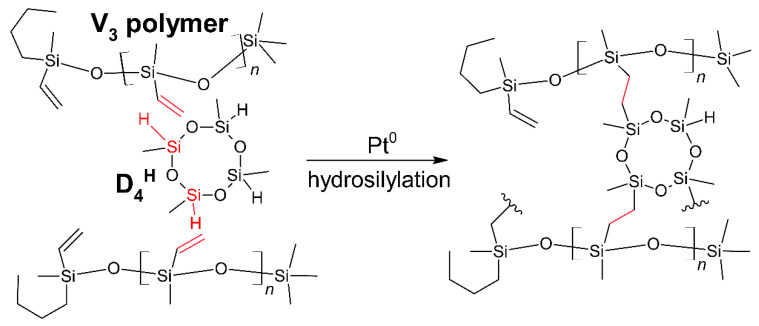
Cross-linking reaction of poly(methylvinylsiloxane)—V_3_ polymer with 1,3,5,7-tetramethylcyclotetrasiloxane (D_4_^H^). The reactive groups in hydrosilylation are marked in red.

Materials studied in this work were prepared by cross-linking of V_3_ polymer with D_4_^H^ in w/o HIPE (Section 2.2, [20]). The reactions were carried out at various molar ratios of Si-Vinyl: Si-H groups in order to obtain various V_3_ polymer cross-linking degrees and various amounts of reactive groups (Si-Vi, Si-H) remaining in the resultant systems. Both these factors seemed to be important for Pd incorporation into the materials as well as for their subsequent ceramization. The chemical structures of the polymer as well as the cross-linking agent that undergo the reaction are presented in Scheme 1.

In our previous work [20], the obtained V_3_ polymer-based polyHIPEs were characterized by equilibrium swelling measurements in tetrahydrofuran (THF), Fourier transform Infrared (FTIR), and ^29^Si Magic Angle Spinning Nuclear Magnetic Resonance (MAS-NMR) spectroscopies as well as SEM observations. In the present investigations, those studies were complemented by Raman spectroscopy and energy dispersive X-ray microanalysis (EDS). To make this work self-contained, however, the swelling results given and discussed in detail in [20] will be briefly summarized here as they are crucial for current investigations. Swelling of the polymer network is related to the polymer’s cross-linking degree that, as mentioned in the ‘Introduction’ (Section 1), influences ceramic yield obtained after pyrolysis of the system.

Swelling experiments showed that (as planned) the samples differed in cross-linking degree. Their swelling decreased—i.e., cross-linking degree increased—as the amount of D_4_^H^ with respect to V_3_ polymer in the experiment increased. It should be noted that swelling of the 2.25V3_1Si-H sample, that exhibited the lowest cross-linking level, was 1.8-times higher than that of the 1V3_1.5Si-H sample, of the highest cross-linking degree [20]. Moreover, swelling of the prepared materials was significantly higher than that of the networks containing methylvinylsiloxane units obtained in the bulk [14] which indicates that their cross-linking degrees were low. This is because, similarly to the previous studies when V_3_ polymer cross-linking was conducted in the bulk [12], participation of all reactive groups of the polymer (with a vinyl group at each Si atom) and D_4_^H^ (with four Si-H groups in the cyclic molecule), due to steric reasons, was improbable. Furthermore, performance of the reactions in HIPE was an additional disadvantage. Polymer cross-linking occurred in thin layers of the continuous phase surrounding high amount of internal phase droplets which must have caused effective contact between the reactive groups difficult.

As described in the ‘Introduction’ (Section 1), cross-linked polysiloxanes are valuable precursors to Si-C-O ceramics. The cross-linked poly(methylvinylsiloxane) materials prepared in this work seemed to be particularly attractive preceramics owing to their porous structure, monolithic nature as well as chemical composition that was expected to result in high ceramic yields. Therefore, the prepared materials were pyrolyzed at 1000 °C in argon atmosphere (Section 2.2). Products of pyrolysis were studied using SEM, FTIR, and Raman spectroscopies.

**Figure 1:**
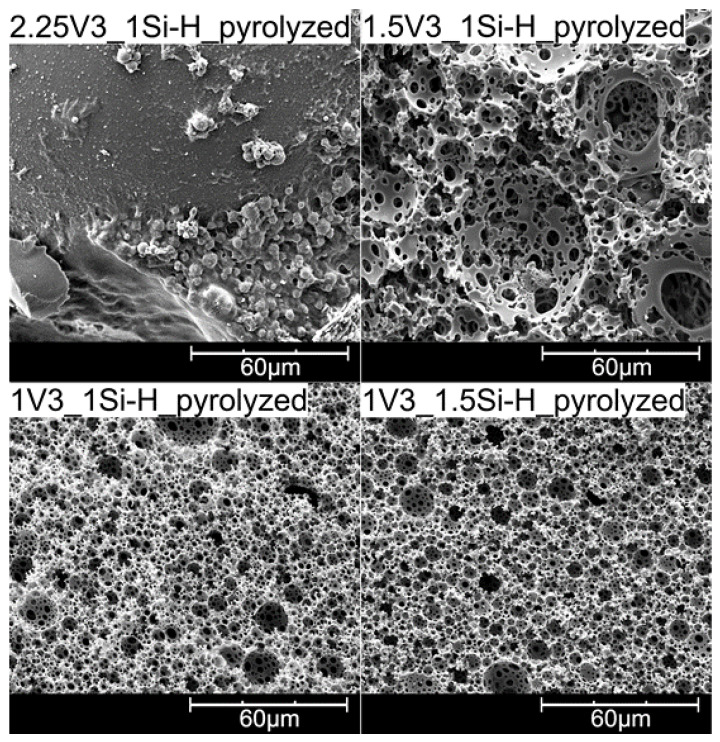
SEM micrographs of polyHIPE materials pyrolyzed at 1000 °C in Ar atmosphere.

In Figure 1, SEM micrographs of the pyrolyzed V_3_ polymer/D_4_^H^ polyHIPE samples are shown. When they are compared with the respective initial ones [20], it becomes clear that pyrolysis did not change the microstructure of the materials. Thus, the pyrolyzed Si-H group rich systems: 1V3_1.5Si-H_pyrolyzed_ and 1V3_1Si-H_pyrolyzed_ showed the morphology typical of polyHIPEs (large macropores interconnected by smaller ones) similarly to their precursors. The pyrolyzed materials of lower contents of Si-H groups: 2.25V3_1Si-H_pyrolyzed_, 1.5V3_1Si-H_pyrolyzed_ contained the non-porous areas on their surfaces, especially numerous in the case of the former sample. Such an effect, observed for the initial samples, was explained by the collapse of HIPEs in the systems caused by too low polymer cross-linking degrees and/or too slow polymer cross-linking process [20].

Table 2 presents the results of EDS quantification obtained from selected microareas in SEM images. In the initial samples, the concentration of carbon decreased while that of silicon grew as the amount of D_4_^H^ in the reaction increased. These changes are consistent with the growing polymer cross-linking degree in the systems. The O:Si atomic ratios greater than one—i.e., the value expected for the perfect polysiloxane network—in the 2.25V3_1Si-H, 1.5V3_1Si-H, and 1V3_1Si-H samples indicate that some of the Si-H groups underwent hydrolysis during formation of polyHIPEs. Upon pyrolysis in all the samples carbon content dropped significantly as the result of hydrocarbon evolution during this process. Simultaneously, silicon and oxygen amounts increased, proving efficient ceramization of the materials. O:Si atomic ratio in the 2.25V3_1Si-H_pyrolyzed_ sample, equal to 2.07—i.e., close to that of silica—suggests that this material was a mixture of SiO_2_ and free carbon. This is not possible as FTIR studies proved unequivocally the presence of Si-C bonds in all the prepared samples (vide infra). Hence, this result shows that the conducted EDS analysis bore some inaccuracy, most probably connected with the standardless method applied (Section 2.3). It should be pointed out, however, that the changes in the element contents observed for the polymer cross-linked with various amounts of D_4_^H^ as well as upon pyrolysis of the materials are consistent with the expected ones. Therefore, the inaccuracy of EDS quantification was not significant for the work.

Pyrolysis of the cross-linked polysiloxanes often leads to Si-C-O materials containing—apart from silicon oxycarbide—free carbon phase [31]. In order to verify if both phases were present in the pyrolyzed V_3_ polymer/D_4_^H^ polyHIPEs, their FTIR and Raman spectra were measured.

**Figure 2:**
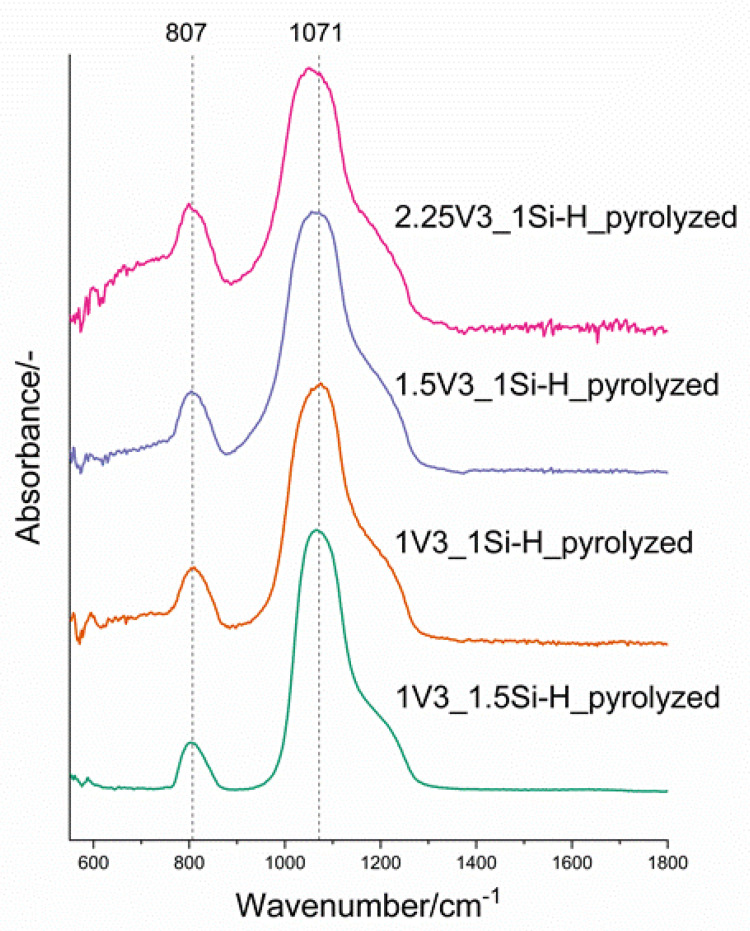
FTIR spectra of the pyrolyzed V_3_ polymer-based polyHIPEs.

**Figure 3:**
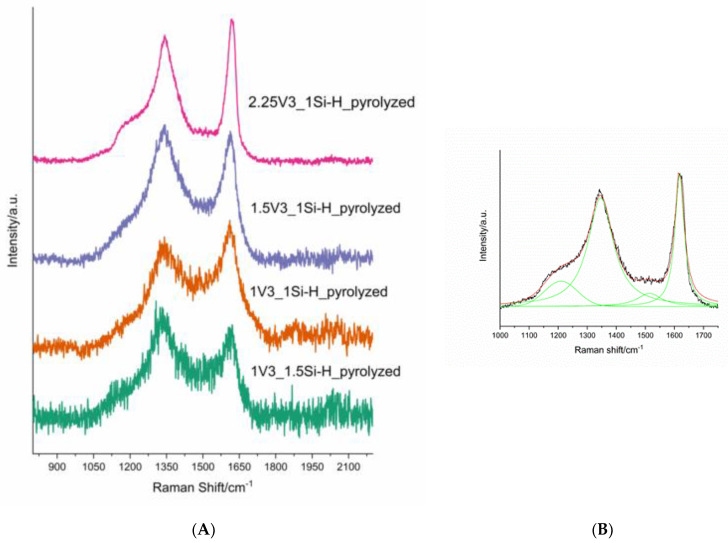
(**A**) Raman spectra of the pyrolyzed polyHIPEs; (**B**) deconvoluted Raman spectrum of the 2.25V3_1Si-H_pyrolyzed_ sample.

FTIR spectra (Figure 2) contain the bands at 1070 cm^−1^ and 807 cm^−1^. The former originates from Si-O-Si asymmetric stretching vibrations, whereas the latter is the superposition of Si-O-Si stretching and Si-C bending bond vibrations [32]. Thus, the spectra confirm that silicon oxycarbides were formed in the systems. When FTIR spectra of the pyrolyzed samples (Figure 2) are compared with those of the initial ones [20], it is seen that—upon pyrolysis—organic groups were totally lost and complete bond rearrangement occurred in the systems. This conclusion can be drawn also based on the Raman spectra. Those corresponding to the materials before pyrolysis (Figure S1) show the bands that are ascribed to various moieties, in particular vinyl and Si-H present in the materials (see the detailed discussion in the Supplementary Information). The Raman spectra of the pyrolyzed samples (Figure 3) contain, in turn, exclusively characteristic set of complex bands in the range between 1100–1700 cm^−1^, originating from free carbon phase. Hence, formation of this phase during pyrolysis is unequivocally detected by Raman spectroscopy.

The complex bands present in the Raman spectra of the pyrolyzed samples were deconvoluted into four components (Figure 3). The two most intense ones, centered at 1346 cm^−1^ and 1617 cm^−1^, are usually referred to as D and G band, respectively [33]. The G band is characteristic for sp^2^ carbon network occurring in graphite, whereas the D band, the so-called disorder-induced band, corresponds to the disordered sp^2^ carbon present at the edges of graphitic domains [33]. The component at 1200 cm^−1^ which gives rise to a shoulder on the D band visible in most of the measured spectra is also connected with the defected graphite structure, namely the presence of sp^3^ carbon (sp^2^C−sp^3^C bonds) and/or double carbon-carbon bonds [34]. The fourth component band, located at 1514 cm^−1^, indicates the existence of amorphous carbon in the samples [34]. In all the Raman spectra, the D band shows high width at half maximum which points to high degree of disorder of the carbon phase generated in the materials. This conclusion is corroborated by higher integral intensity of the D than that of the G band in all the cases (Table 2). Low ordering of the carbon phase formed in the systems can be explained by relatively low temperature of pyrolysis of the precursors (1000 °C). More ordered graphitic structure is usually developed in the Si-C-O materials obtained at higher temperatures [35,36]. Carbon domain sizes, calculated according to [33] in all the pyrolyzed V_3_ polymer/D_4_^H^ polyHIPE samples, were in the range of 6–8 nm (Table 2).

### 3.2. Pd-Containing Materials: XRD, SEM, and Spectroscopic Studies

As aforementioned, non-porous polysiloxane networks containing Si-H groups reduce Pd^2+^ ions in tetrahydrofuran solution to form Pd/polymer nanocomposites [14]. Similar effect was found for V_3_ polymer-based polyHIPEs [20]. The studies discussed in the previous section showed, in turn, that pyrolysis of these macroporous materials leads to the Si-C-O ceramics of the preserved microstructure. Thus, pyrolysis in an inert atmosphere of the V_3_ polymer/D_4_^H^ polyHIPEs treated with Pd^2+^ ions in THF solution seemed to be a convenient way to obtain Pd/porous Si-C-O material nanocomposites.

To prepare Pd-containing Si-C-O materials the obtained V_3_ polymer/D_4_^H^ polyHIPEs were stirred in the THF solution of Pd(OAc)_2_ and, after washing with THF and drying, were subjected to pyrolysis in Ar atmosphere at 1000 °C (Section 2.2). Pd-containing samples before and after pyrolysis were examined by XRD to check if metallic Pd existed in the systems. Additionally, they were studied by SEM using back-scattered electrons (BSE) detector and their IR and Raman spectra were measured.

**Figure 4:**
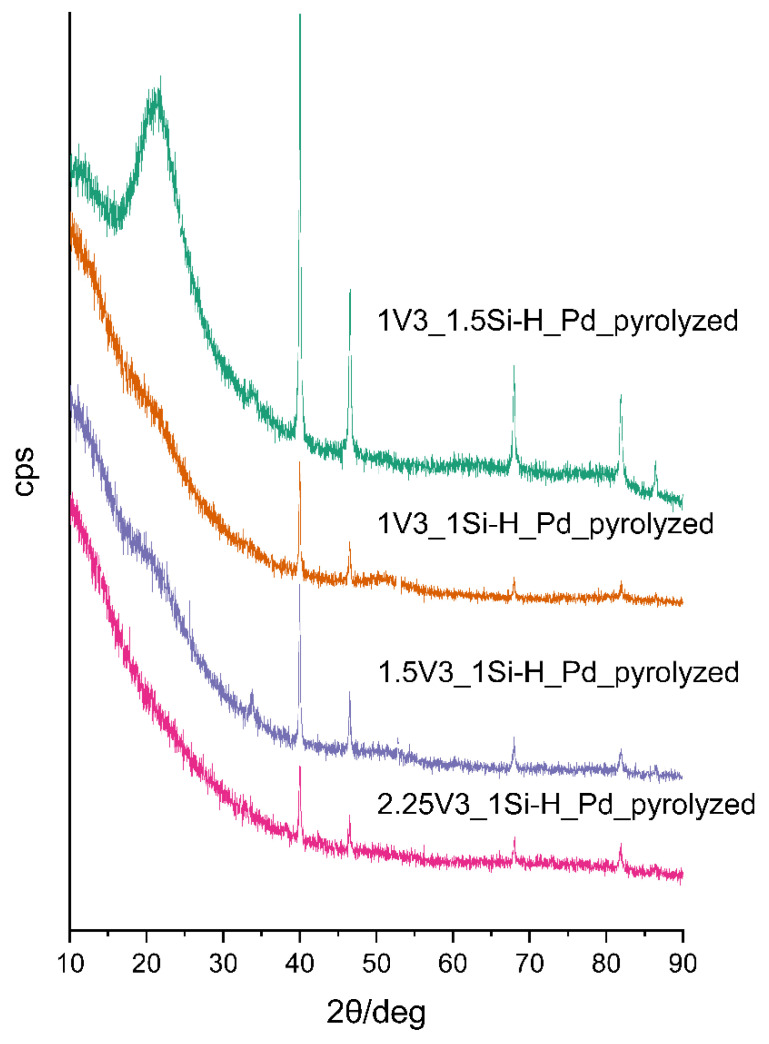
XRD patterns of the pyrolyzed Pd-containing V_3_ polymer-based polyHIPE.

XRD showed that Pd nanoparticles were formed in all the initial systems [20], whereas Figure 4 presents XRD patterns of the pyrolyzed Pd-containing samples. In all cases, the reflection at 2θ angle equal to 39.5° which corresponds to the most intense diffraction line of Pd fcc crystalline lattice (Pd (111) plane) [37] can be seen. All the patterns also contain distinct reflections at 2θ = 46.5°, 68.0° 81.9°, and 86.4°, assigned to Pd(200), Pd(220), Pd(311), and Pd(222) planes, respectively [37]. Hence, XRD shows that metallic Pd—initially dispersed in the polymer matrix—is retained in the pyrolyzed materials.

The reflections corresponding to crystalline Pd occurring in the XRD patterns of the materials before pyrolysis are broad and of low intensity [20]; in contrast, they are narrow and exhibit high intensity in the XRD traces of the pyrolyzed samples (Figure 4). Narrowing of the reflections after pyrolysis indicates sintering of Pd clusters during this process. Calculations performed using Scherrer equation (Section 2.3, Equation (2)) gave the mean crystallite sizes in the range of 4.9–8.1 nm and 35.4–47.7 nm in the samples before and after pyrolysis, respectively (Table 3). Pyrolysis caused 4.7- to 8.5-fold increase in the mean sizes of Pd crystallites.

Growth in the intensities of the Pd lines in the XRD patterns is related to the increased metal contents in the pyrolyzed materials in comparison with the materials subjected to pyrolysis. The initial samples contained various amounts of Pd (Table 3) and—as will be shown in Section 3.3—they also differed in ceramic yields (Table 4). In effect, Pd contents in the 1.5V3_1Si-H_Pd_pyrolyzed_ and 1V3_1Si-H_Pd_pyrolyzed_ systems were comparable, in the 1V3_1.5Si-H_Pd_pyrolyzed_ lower and in the 2.25V3_1Si-H_Pd_pyrolyzed_ the lowest (Table 3). Inspection of the measured XRD patterns, however, allows us to conclude that intensities of the Pd peaks corresponding to metal present in the pyrolyzed samples (Figure 4) are not related to the total Pd amounts in the systems. The most intense Pd peaks are seen in the pattern of the 1V3_1.5Si-H_Pd_pyrolyzed_ material even though Pd content in it was not the highest (Table 3). The least intense Pd peaks are observed in the XRD pattern of the 1V3_1Si-H_Pd_pyrolyzed_ sample with metal content close to that found for the 1.5V3_1Si-H_Pd_pyrolyzed_ material. This implies that fractions of metallic Pd in the systems were different, i.e., reduction of Pd^2+^ ions in Pd(OAc)_2_ solution in THF by the initial polyHIPEs was not similarly efficient in all the cases. This may be due to, revealed by Raman spectroscopy (see discussion in the Supplementary Information, Section 1S), differences in the amounts of Si-H groups remaining in the networks after polymer cross-linking and/or differences in their accessibility for Pd^2+^ ions from the solution in various systems. It is worth noting that catalytic studies of the Pd-containing V_3_ polymer-based polyHIPEs also pointed to different amounts of metallic Pd in the systems [20].

**Figure 5:**
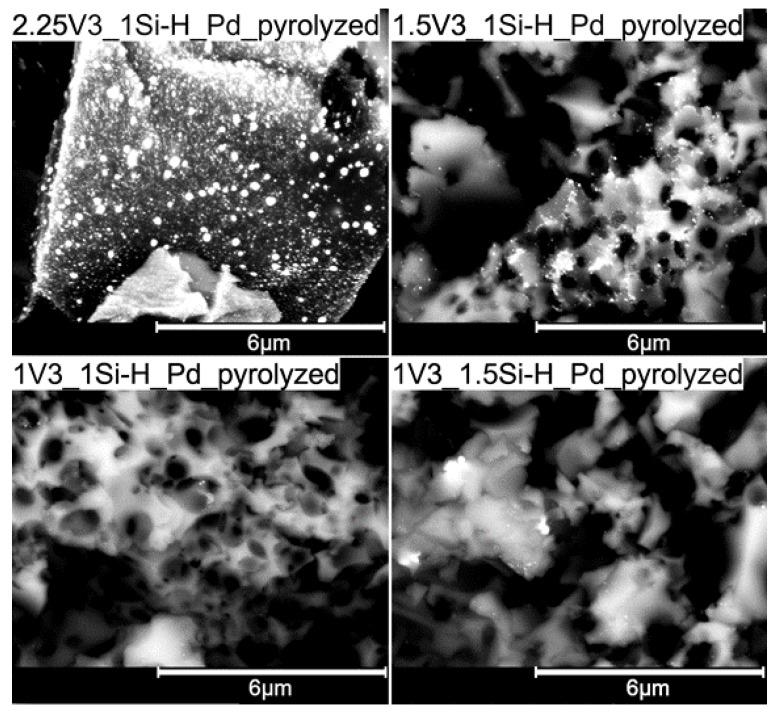
BSE images of Pd-containing samples after pyrolysis.

BSE images (Figure 5) show that the pyrolyzed materials contained large Pd agglomerates whose distribution and numbers on their surfaces were different. The highest number of agglomerated Pd particles is visible in the BSE image of the 2.25V3_1Si-H_Pd_pyrolyzed_ sample. Lower numbers of Pd agglomerates are seen in the BSE images of the 1.5V3_1Si-H_Pd_pyrolyzed_ and 1V3_1.5Si-H_Pd_pyrolyzed_ polyHIPEs, possibly related to their more porous morphology. The Pd agglomerates are practically absent in the 1V3_1Si-H_Pd_pyrolyzed_ system which—in view of the XRD results—may be due to the lowest content of metallic Pd in it. Simultaneously, the uniform, bright BSE image indicates that very small Pd nanoparticles, not resolved under our experimental conditions, were present and evenly distributed on the surface of this material. Importantly, BSE images of the pyrolyzed samples do not show differences with respect to those of the starting Pd-containing polyHIPEs [20]. This means that, even though the sizes of Pd crystallites detected by XRD grew, the numbers and sizes of Pd agglomerates visible in SEM did not change upon pyrolysis.

Raman spectra of the Pd-containing materials before pyrolysis are presented and discussed in the Supplementary Information (Figure S2, Section 2S). FTIR and Raman spectra of the pyrolyzed samples (not shown) are unchanged with respect to those measured for the pyrolyzed materials without incorporated Pd (Figure 2 and Figure 3). This demonstrates that, after pyrolysis, Pd particles were dispersed within matrices composed of silicon oxycarbide and free carbon phases. Mean sizes of carbon domains calculated based on Raman spectra (Table 3), however, did not change systematically as compared to the pyrolyzed systems without Pd (Table 2).

### 3.3. Thermal Investigations

The initial V_3_ polymer/D_4_^H^ polyHIPEs and those with incorporated Pd particles were examined by thermogravimetry (TG, Section 2.3). The studies were first aimed at evaluating how the polymer cross-linking degree in the starting polyHIPEs influenced their thermal properties, and then at determining if these properties were changed upon incorporation of Pd into the systems.

**Figure 6:**
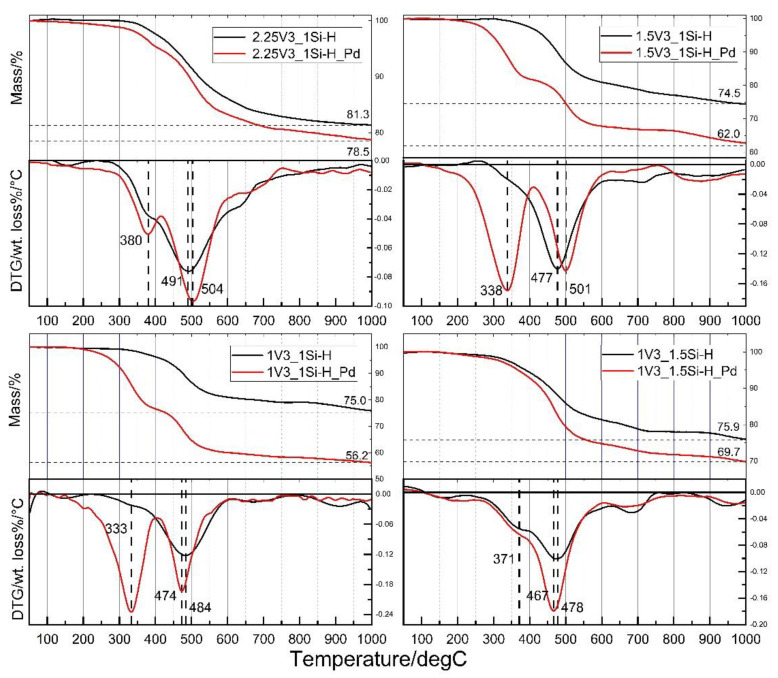
TG and derivative TG (DTG) curves obtained for the initial and Pd-containing V_3_ polymer-based polyHIPEs studied in the work.

TG plots (Figure 6) clearly show that regardless of the Si-Vinyl: Si-H group molar ratio used in their preparation and hence their cross-linking densities, all initial polyHIPEs began to decompose near 300 °C. Their thermal stability at higher temperatures was, however, different. This can be judged by the values of temperature at which the analyzed samples lost 5% (T_5_) and 10% (T_10_) of their starting masses (Table 3). T_5_ varies in a systematic way: it decreases as the polymer cross-linking degree grows. T_10_ in turn is the highest for the 2.25V3_1Si-H material and the lowest for the 1V3_1.5Si-H one, showing respectively the lowest and the highest cross-linking degree (Section 3.1). T_10_ values observed for the specimens of intermediate polymer cross-linking densities (1.5V3_1Si-H and 1V3_1Si-H) are practically the same. Importantly, however, the difference between T_10_ and T_5_ values, and hence thermal stability in this temperature range, is the same for the 2.25V3_1Si-H and 1V3_1.5Si-H materials (Table 3). This difference—and thermal stability—is lower for the 1V3_1Si-H, and the lowest for the 1.5V3_1Si-H one (Table 3). It can be considered that the values of T_5_ reflect the behavior of the studied polyHIPEs in lower, while the T_10_ and T_5_ difference—in higher temperature ranges. Therefore, the described results indicate that the amount of the cross-linker introduced into the systems mainly influenced the first stages of their thermal decomposition. As the consequence of their thermal behavior, the highest ceramic yield at 1000 °C was observed for the 2.25V3_1Si-H material (81.3 mass %, Table 4); ceramic yields found for the 1.5V3_1Si-H, 1V3_1Si-H, and 1V3_1.5Si-H ones were close (74.5–75.9 mass %, Table 4).

Incorporation of palladium into the materials resulted in the decrease in their thermal stability: decomposition of the Pd-containing samples began at temperatures lower by 100–150 °C than those of the respective initial ones; the difference was less significant (ca. 30 °C) in the case of the 1V3_1.5Si-H and 1V3_1.5Si-H_Pd materials (Figure 6). T_5_ and T_10_ values also decreased for all the systems with introduced Pd when compared to the respective starting ones (Table 3). It should be noted that Pd had an extremely high impact on thermal properties of the 1.5V3_1Si-H and 1V3_1Si-H polyHIPEs for which the highest shifts in the T_5_ and T_10_ values were observed. Pd influence on the 1V3_1.5Si-H material was the lowest. Additionally, it is worth noting that, among the Pd-containing samples, the 2.25V3_1Si-H_Pd—similarly to the 2.25V3_1Si-H among the starting ones—was characterized by the highest thermal stability as it showed the highest T_5_ and T_10_ as well as the largest difference between their values (Table 4). Due to lower thermal stability, all the Pd-containing polyHIPEs gave lower ceramic yields at 1000 °C than the respective starting ones (Table 4).

From DTG curves (Figure 6) it is clear that in most cases incorporation of Pd modifies the mechanism of thermal decomposition of the studied polyHIPEs. Intensification of the first (2.25V3_1Si-H_Pd vs. 2.25V3_1Si-H materials) or appearance of a new (1.5V3_1Si-H_Pd vs. 1.5V3_1Si-H and 1V3_1Si-H_Pd vs. 1V3_1Si-H materials) decomposition step at lower temperature is observed (Figure 6, Table 4). DTG plots of the 1V3_1.5Si-H_Pd and 1V3_1.5Si-H samples are the exception: they are similar which further confirms that the impact of Pd on thermal behavior of the 1V3_1.5Si-H polyHIPE is the least significant.

All these findings can be rationalized taking into account our previous studies on application of poly(vinylsiloxanes) as precursors to Si-C-O ceramics as well as the literature data. From our experience, it follows that thermal decomposition of poly(vinylsiloxanes) cross-linked by hydrosiloxanes under inert atmosphere starts with the cleavage of Si-H bonds preserved in the systems [14,38]. We also found that liquid V_3_ polymer solidifies in Ar atmosphere at 245 °C due to thermal cross-linking of its vinyl groups [38]. According to other [7,35,36,39,40,41] and our investigations [14], in the temperature range of 300–600 °C polysiloxane networks undergo the Si-H/Si-O, Si-C/Si-O, and Si-O/Si-O bond redistributions. These reactions are accompanied by the evolution of volatile silicon compounds of relatively high molecular masses, which—if these processes take place—leads to significant mass loss of the sample pyrolyzed in this temperature range. Si-H/Si-O redistributions occur first, at ca. 300 °C, then Si-C/Si-O—at ca. 500 °C, and finally Si-O/Si-O—in the range of 500–600 °C [39].

Decrease in thermal stability of the starting polyHIPEs with the increase in their cross-link density at initial stages (vide supra) can be explained by the growing contents of thermally unstable Si-H groups in the materials revealed by Raman spectroscopy (Supplementary Information, Section 1S). The highest ceramic yield at 1000 °C found for the 2.25V3_1Si-H polyHIPE (Table 4) may be, in turn, related to low amount of Si-H groups as well as to thermal cross-linking of polymer chains with the participation of vinyl groups, possible due to low initial cross-link density and high flexibility of polymer chains retained in this system. Both factors—i.e., low amount of Si-H groups and polymer thermal cross-linking—limit the generation of volatile Si compounds, thus increasing ceramic yield.

Unambiguously, as shown by TG studies (changes in thermal behavior of the materials at temperatures below 600 °C), incorporation of Pd modifies the course of thermal redistributions of bonds at Si atoms in the studied polyHIPEs. In particular, those occurring at the lowest temperature are intensified (see the DTG curves in Figure 6 and their discussion above) leading to lowering in ceramic yields in the systems. As can be judged by the lowest impact of Pd on thermal behavior of the 1V3_1.5Si-H sample, higher initial polymer cross-linking degree is advantageous. Similarly, low effect of Pd is observed when polymer cross-linking occurs during heat treatment of the material as in the case of the 2.25V3_1Si-H polyHIPE.

The conclusion on modification of Si bond redistributions is consistent with our previous report on the influence of Pd on thermal properties of non-porous polysiloxane networks [14]. However, in those systems, the opposite effects were observed—i.e., retarded or less intense exchange of the bonds at Si atoms. This can be explained by the aforementioned (Section 3.1) higher initial cross-linking levels of the polymers studied in that work.

Thus, our work sheds new light on the complex influence of Pd on thermal degradation of preceramic polysiloxane networks containing Si-H groups. It clearly shows that this effect is strongly related to the amount of Si-H groups and to the polymer cross-linking degree in the system.

## 4. Conclusions

In the work, poly(methylvinylsiloxane)—i.e., V_3_ polymer-based polyHIPEs and V_3_ polymer-based polyHIPE–Pd systems—were obtained and studied as precursors to Si-C-O and Si-C-O-Pd ceramics. The conducted investigations inform the following conclusions:

1. Pyrolysis of the V_3_ polymer-based polyHIPEs performed in Ar atmosphere at 1000 °C leads to Si-C-O materials composed of silicon oxycarbide and free carbon phases; the microstructure of the precursors is preserved in this process, ceramic yields are high, in the range of 74.5–81.3 mass %.
2. V_3_ polymer-based polyHIPEs with incorporated metallic Pd nanoparticles, after pyrolysis carried out in Ar atmosphere at 1000 °C give Si-C-O ceramics, containing silicon oxycarbide and free carbon phases, with incorporated metallic Pd particles. Upon pyrolysis, sintering of the initial Pd nanoparticles occurs.
3. Incorporation of Pd significantly affects thermal properties of the V_3_ polymer-based polyHIPEs. The materials with introduced Pd particles show lower thermal stability and give lower ceramic yields at 1000 °C (56.2–78.5 mass %) than the initial ones. Pd mainly influences the mechanism of bond redistributions at Si atoms that occur in the temperature range between 300 and 600 °C. The extent to which Pd modifies thermal properties of the V_3_ polymer-based polyHIPE is related to polymer cross-linking degree in the material.

To summarize, the work shows that polysiloxane-based polyHIPEs can be applied as precursors to Si-C-O ceramics of controlled microstructure. They can be also used as matrices for incorporation of metallic particles and transformed into Si-C-O/metal composite materials. In our opinion, these systems are of great potential for both further investigations and applications. This is due to advantageous properties of polysiloxane networks and Si-C-O ceramics which exhibit high chemical and (especially Si-C-O materials) high thermal stability. Therefore, such systems with dispersed metallic particles of catalytic properties— like Pd studied in this work—can be used as catalysts in a wide range of chemical processes, even those involving aggressive reactants and/or high temperatures.

## Figures and Tables

**Table 1:** Amounts of components used in the preparation of the studied polyHIPEs.

Sample	Continuous Phase					Internal Phase
	V_3_ Polymer (g)	D_4_^H^ (g)	DBE-224 (g)	Chlorobenzene (g)	Catalyst (μL)	0.02M NaCl_(aq)_ (g)
2.25V3_1Si-H	1	0.31	0.33	0.41	7	9.32
1.5V3_1Si-H	1	0.47	0.36	0.46	7	10.43
1V3_1Si-H	1	0.70	0.43	0.53	7	12.10
1V3_1.5Si-H	1	1.13	0.53	0.67	7	15.15

**Table 2:** EDS quantification of C, O, and Si concentrations in selected microareas, ratios of integral intensity of the D (I_D_) and G (I_G_) bands in the Raman spectra and mean carbon domain sizes in the pyrolyzed samples calculated based on Raman spectra of the pyrolyzed samples.

Sample	Concentration/at %			C:O:Si Atomic Ratio (EDS)	I_D_/I_G_ Ratio	Mean Carbon Domain Size in the Pyrolyzed Sample/nm
	C	O	Si			
2.25V3_1Si-H 2.25V3_1Si-H*_pyrolyzed_*	52.08 10.40	28.51 60.45	19.42 29.15	2.68:1.47:1 0.35:2.07:1	− 2.48	− 7
1.5V3_1Si-H 1.5V3_1Si-H*_pyrolyzed_*	50.54 6.84	27.14 60.80	22.33 32.36	2.26:1.22:1 0.21:1.88:1	− 3.22	− 6
1V3_1Si-H 1V3_1Si-H*_pyrolyzed_*	50.32 5.26	27.31 58.56	22.37 36.18	2.25:1.22:1 0.14:1.62:1	− 2.48	− 8
1V3_1.5Si-H 1V3_1.5Si-H*_pyrolyzed_*	47.58 8.05	27.07 57.97	25.35 33.98	1.88:1.07:1 0.24:1.71:1	− 2.71	− 7

**Table 3:** Pd contents and mean crystallite sizes in the pyrolyzed samples, ratios of integral intensity of the D (I_D_) and G (I_G_) bands in the Raman spectra and carbon domain sizes in the Pd-containing pyrolyzed samples calculated based on Raman spectra of the pyrolyzed samples.

Sample	Pd Content/wt % ^1^	Mean Pd Crystallite Size/nm ^2^	I_D_/I_G_ Ratio	Mean Carbon Domain Size/nm
2.25V3_1Si-H_Pd_pyrolyzed_	1.39	36.9	2.34	8
1.5V3_1Si-H_Pd_pyrolyzed_	2.74	42.7	1.86	10
1V3_1Si-H_Pd_pyrolyzed_	2.66	47.7	3.20	6
1V3_1.5Si-H_Pd_pyrolyzed_	2.03	35.4	2.06	9

^1^ Pd contents in the pyrolyzed samples were calculated taking into account ceramic yields given in Table 4 and the content of Pd in the initial sample, determined by X-ray fluorescence (XRF). Pd contents in the samples subjected to pyrolysis were as follows: 2.25V3_1Si-H_Pd—1.09 wt %, 1.5V3_1Si-H_Pd—1.70 wt %, 1V3_1Si-H_Pd—1.49 wt %, 1V3_1.5Si-H_Pd—1.42 wt % [20]; ^2^ Calculated based on XRD patterns using Scherrer equation (Section 2.3, Equation (2)). Mean Pd crystallite sizes in the samples subjected to pyrolysis were as follows: 2.25V3_1Si-H_Pd—7.8 nm, 1.5V3_1Si-H_Pd—8.1 nm, 1V3_1Si-H_Pd—5.6 nm, 1V3_1.5Si-H_Pd—4.9 nm [20].

**Table 4:** Values of 5% mass loss (T_5_), 10% mass loss (T_10_), differences between T_10_ and T_5_ (T_10_−T_5_), ceramic yields at 1000 °C, and temperatures of the fastest decomposition of the analyzed samples.

Sample	T_5_/°C	T_10_ (T_10_−T_5_)/°C	Ceramic Yield at 1000 °C/mass%	Temperatures of the Fastest DecomPosition/°C
2.25V3_1Si-H 2.25V3_1Si-H_Pd	452 410	521 (69) 493 (83)	81.3 78.5	380 (sh ^1^), 491 380, 504
1.5V3_1Si-H 1.5V3_1Si-H_Pd	440 297	475 (35) 330 (33)	74.5 62.0	477 338, 501
1V3_1Si-H 1V3_1Si-H_Pd	426 280	477 (51) 313 (33)	75.0 56.2	484 333, 474
1V3_1.5Si-H 1V3_1.5Si-H_Pd	391 368	459 (68) 433 (65)	75.9 69.7	371 (sh ^1^), 478 371 (sh ^1^), 467

^1^ “sh” stands for “shoulder”.

## Data Availability

The data is contained within the article and Supplementary Material.

## Supplementary Materials

The following are available online at https://www.mdpi.com/article/10.3390/ma14195661/s1, Figure S1: Raman spectra of the starting V_3_ polymer-based polyHIPEs studied in the work, Figure S2: Raman spectra of the materials with incorporated Pd before pyrolysis.
