# Supplementary material for: Porous Materials Based on Poly(methylvinylsiloxane) Cross-Linked with 1,3,5,7-Tetramethylcyclotetrasiloxane in High Internal Phase Emulsion as Precursors to Si-C-O and Si-C-O/Pd Ceramics
Source: Materials (Basel). 2021 Sep 29;14(19):5661. doi: 10.3390/ma14195661 (PMC8510497; doi:10.3390/ma14195661)
Supplement: Supplementary file 1 [file materials-14-05661-s001.zip › materials-1336734-supplementary.pdf]

materials

Supplementary Materials

Porous Materials Based on Poly(methylvinylsiloxane) Cross-Linked with 1,3,5,7-Tetramethylcyclotetrasiloxane in High Internal Phase Emulsion as Precursors to Si-C-O and Si-C-O/Pd Ceramics

1S. Raman spectra of the initial, Pd-free polyHIPEs

Chemical composition of the prepared polyHIPEs before pyrolysis was characterized by Raman spectroscopy. This technique was selected as, in comparison to the previously applied FTIR spectroscopy [1], it shows higher sensitivity to hydrocarbon, in particular vinyl groups existing in the studied systems. Figure S1 presents Raman spectra of the prepared cross-linked V_3 polymer samples. The bands characteristic for vinyl groups, located at 3060 cm^{-1} (=CH_2 asymmetric stretching [2]) and at 1596 cm^{-1} (C=C) stretching [2]) are distinctly seen. Their intensities drop in the spectra of the samples obtained with increasing amounts of the cross-linking agent which proves that conversion of polymer's vinyl groups in the reaction grew as the concentration of D_4^H in the emulsion increased. This is in line with the increase in the polymer cross-linking degree found by swelling experiments. The band attributed to Si-H, i.e. the other groups participating in the polymer's cross-linking process, located at 2164 cm^{-1} (Si-H stretching [2]) is weak and broad. It is, however, well resolved in the spectra of the 1V3_1.5Si-H and 1V3_1Si-H samples, prepared with higher amounts of the cross-linking agent. This shows that higher fractions of unreacted Si-H groups remained in the materials when higher amounts of D_4^H were applied in their preparation. It should be noted that the most intense bands in all the spectra, at 2904 cm^{-1} and 2970 cm^{-1} arise from symmetric and asymmetric C-H (sp^3) stretching [2], respectively and that at 1408 cm^{-1} is the result of CH_3 asymmetric deformation [2]. The Si-O-Si symmetric stretching band is located at 502 cm^{-1} [2]. Thus, Raman spectra showed that both types of reactive groups, i.e. vinyl and Si-H, were preserved in the materials; their amounts depended on polymer crosslinking degree.

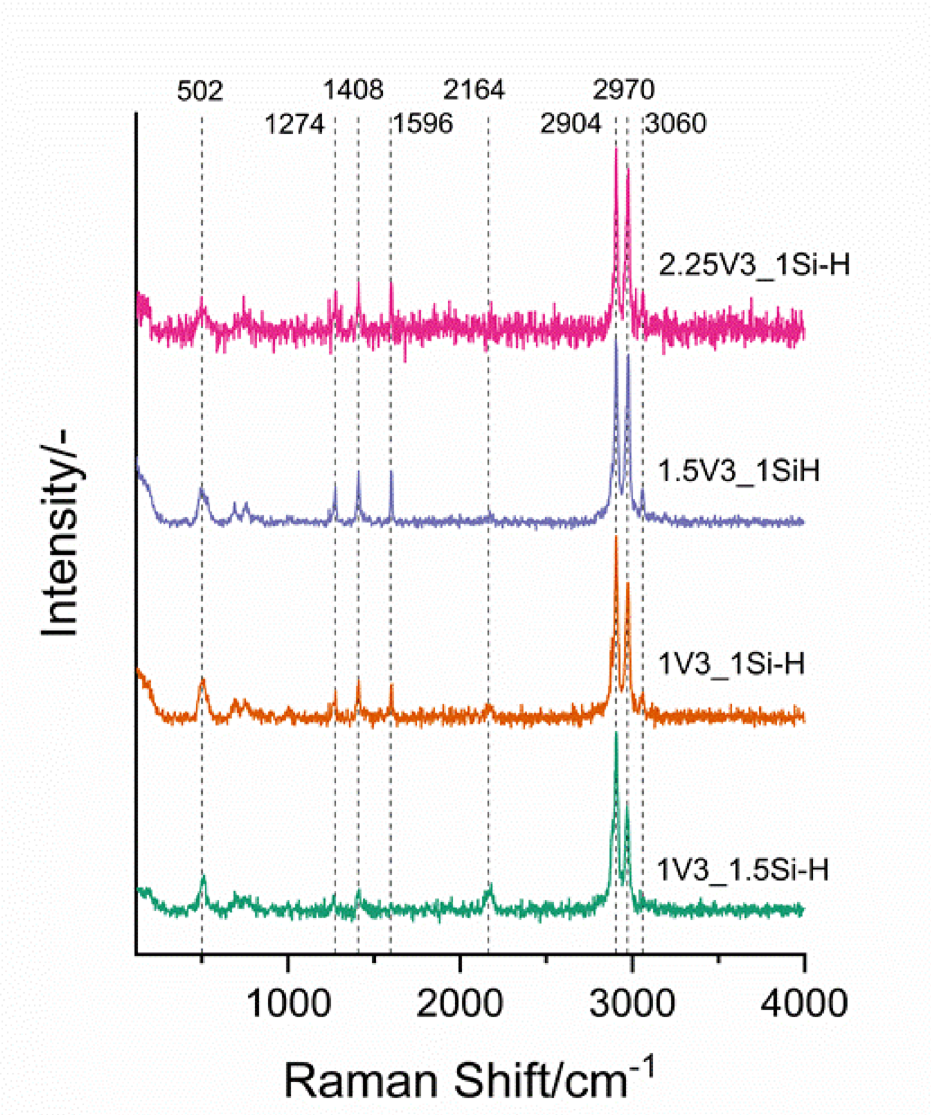

Figure S1. Raman spectra of the starting V_3 polymer-based polyHIPEs studied in the work.

2S. Raman spectra of the initial, Pd-containing polyHIPEs

Raman spectroscopy was used to determine changes in the chemical composition of the starting V_3 polymer/D_4^H polyHIPEs after treatment with $Pd(OAc)_2$ solution. In the spectra of the Pd-containing materials (Figure S2), similarly to those of the initial samples (Figure S1), the bands corresponding to vinyl groups (3060 cm^{-1} and 1596 cm^{-1}), C-H (sp^3) bonds (2904 cm^{-1}, 2970 cm^{-1}, 1408 cm^{-1}, 1274 cm^{-1}), Si-O-Si bridges (502 cm^{-1}) and Si-H bonds (2164 cm^{-1}) are distinguished; their positions are the same as those in the initial spectra. Thus, chemical structure of the polymer matrix stayed intact upon Pd incorporation. Importantly, changes in the intensity of the band at 2164 cm^{-1} originating from the Si-H bonds should be noted. This band, of low intensity in the spectrum of 1.5V3_1Si-H material, is absent in the spectrum of 1.5V3_1Si-H_Pd sample. Lowering in the intensity of this band when compared with the starting ones can be noticed in the spectra of 1V3_1Si-H_Pd and 1V3_1.5Si-H_Pd systems (Figure S2). Hence, Raman spectra confirm that reduction of Pd^{2+} ions proceeded at the expense of Si-H groups. However, not all of Si-H groups present in the systems were consumed in the process.

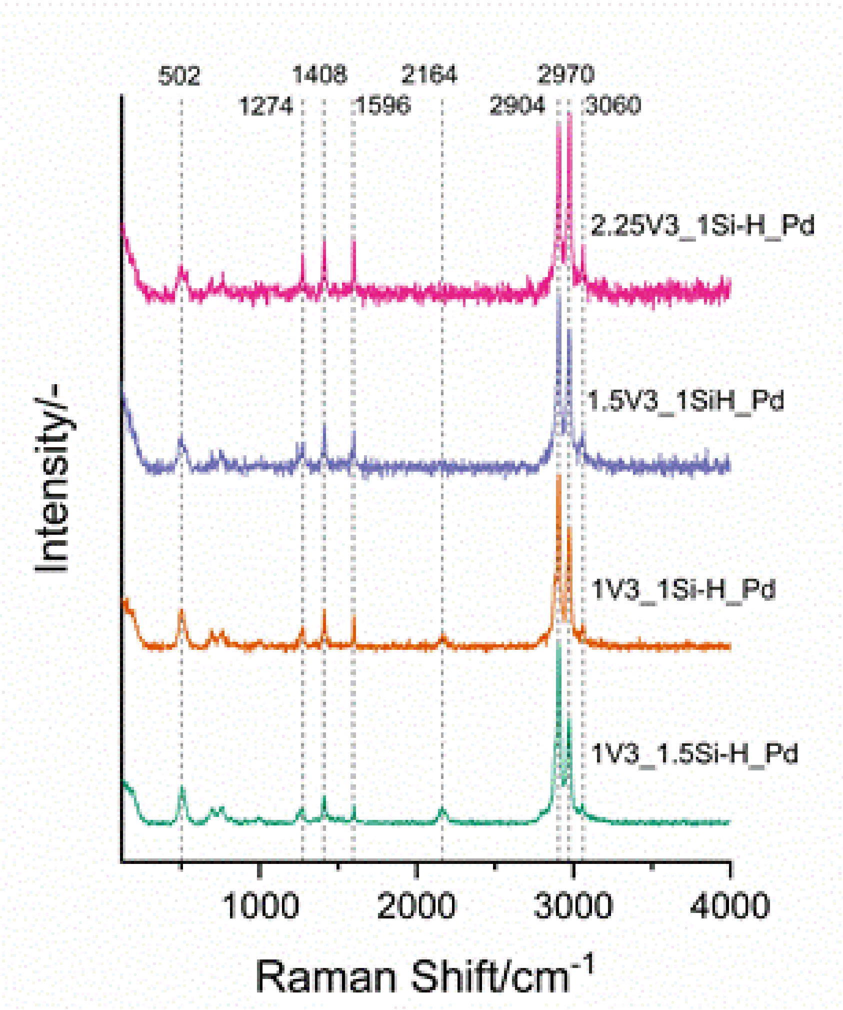

Figure S2. Raman spectra of the materials with incorporated Pd before pyrolysis.

References

1. Mrówka J.; Gackowski M.; Lityńska-Dobrzyńska, L.; Bernasik, A.; Kosydar, R.; Drelinkiewicz, A.; Hasik, M. Poly(methylvinylsiloxane)-Based High Internal Phase Emulsion-Templated Materials (polyHIPEs)—Preparation, Incorporation of Palladium, and Catalytic Properties, *Ind. Eng. Chem. Res.* **2020**, *59*, 19485–19499. https://doi.org/10.1021/acs.iecr.0c03429.
2. Socrates, G. *Infrared and Raman Characteristic Group Frequencies: Tables and Charts*. 3rd Edition; Wiley: New York, NY, USA, 2004.
